# Enhancing Air Traffic Control Communication Systems with Integrated Automatic Speech Recognition: Models, Applications and Performance Evaluation

**DOI:** 10.3390/s24144715

**Published:** 2024-07-20

**Authors:** Zhuang Wang, Peiyuan Jiang, Zixuan Wang, Boyuan Han, Haijun Liang, Yi Ai, Weijun Pan

**Affiliations:** College of Air Traffic Management, Civil Aviation Flight University of China, Guanghan 618307, China; wangzhuang@cafuc.edu.cn (Z.W.); peiyuan_jiang@163.com (P.J.); fannyw821@gmail.com (Z.W.); hanregulu@gmail.com (B.H.); navyliang@cafuc.edu.cn (H.L.); aiyi@cafuc.edu.cn (Y.A.)

**Keywords:** air traffic control, speech communication, automatic speech recognition

## Abstract

In air traffic control (ATC), speech communication with radio transmission is the primary way to exchange information between the controller and the pilot. As a result, the integration of automatic speech recognition (ASR) systems holds immense potential for reducing controllers’ workload and plays a crucial role in various ATC scenarios, which is particularly significant for ATC research. This article provides a comprehensive review of ASR technology’s applications in the ATC communication system. Firstly, it offers a comprehensive overview of current research, including ATC corpora, ASR models, evaluation measures and application scenarios. A more comprehensive and accurate evaluation methodology tailored for ATC is proposed, considering advancements in communication sensing systems and deep learning techniques. This methodology helps researchers in enhancing ASR systems and improving the overall performance of ATC systems. Finally, future research recommendations are identified based on the primary challenges and issues. The authors sincerely hope this work will serve as a clear technical roadmap for ASR endeavors within the ATC domain and make a valuable contribution to the research community.

## 1. Introduction

The development of civil aviation is essential for both national economic growth and international expansion. One critical aspect of civil aviation is air traffic control (ATC), a complex and dynamic system emphasizing flight safety and efficiency, which is always a hot research topic. According to the Federal Aviation Administration (FAA) workload forecast, operations at airports, approaches, and routes are expected to increase rapidly from 2020 to 2040 [1], reflecting the growth and dynamism of the aviation industry. Air traffic mainly relies on the manual command provided by air traffic controllers (ATCos). However, the high-density airspace environment places continuous and significant demands on ATCos, often leading to long periods of saturated workloads. This unrelenting pressure underscores the need for ongoing research and development to enhance ATC systems and alleviate the challenges faced by ATCos.

During an ATC procedure, ATCos transmit ATC commands using very high frequency (VHF) radiotelephony, and it is imperative for the receiving pilots to accurately replicate these commands. Within a communication channel are one ATCo and multiple pilots conducting frequent and rapid speech, which requires ATCos and pilots to understand each other’s intentions accurately and efficiently. Communication between ATCos and pilots is characterized by stringent standards, a fast-paced environment, and the potential for noise and interference during conversations. Occasional aviation incidents still occur due to errors in radiotelephony communication, like inaccurate repetition, misunderstandings, and incomplete content. Using automatic speech recognition (ASR) can effectively address this issue.

ASR, which emerged in the 1950s, is a transformative technology that transcribes spoken language into text, enabling computers to comprehend and process human speech [2]. The development of artificial intelligence, specifically deep learning, has dramatically enhanced ASR by implementing deep neural networks DNNs [3]. A breakthrough has been made using the end-to-end (E2E) model, a single network that directly converts the input speech sequence into an output text sequence [4]. Modern ASR represents a dynamic and interdisciplinary field at the intersection of computer science, linguistics, signal processing, and artificial intelligence. It has not only experienced remarkable advancements but has also found extensive applications across diverse domains [5].

**Figure 1:**
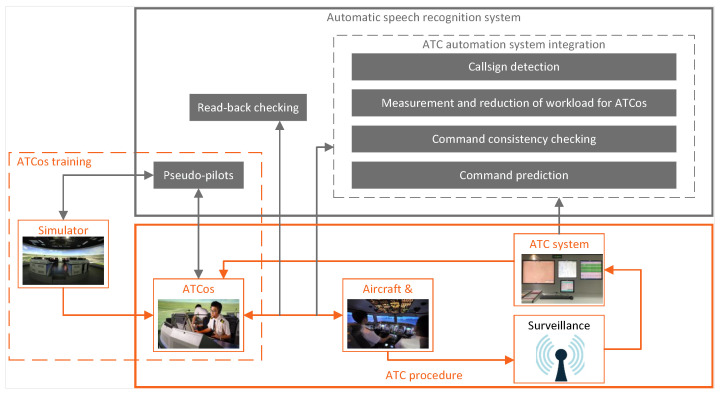
Roles of ASR in the ATC procedure.

ASR technology holds immense promise within the realm of ATC by offering the potential to elevate communication precision, alleviate ATCo workloads, and enhance overall situational awareness, thus bolstering the safety of air traffic. One of its critical applications is to check the correctness of pilots’ repetitions. Additionally, a proficiently trained ASR system can serve as multiple pseudo-pilots in ATCos training. Furthermore, ASR integration with existing ATC automation systems can facilitate tasks such as callsign detection, measurement and reduction of workload for ATCos, command consistency checking and command prediction. These multifaceted capabilities underscore ASR’s potential to revolutionize and optimize various facets of ATC. The applications of ASR in the ATC domain are shown in Figure 1.

Although ASR has made significant advances across various domains, its exploratory use in ATC has revealed that while using ASR to assist ATCos shows some effectiveness, it does not overall reduce ATCos’ workload. Instead, it introduces additional burdens, primarily due to several reasons:

- Specialized vocabulary: Numerous rules and measures to regulate the pronunciation of homophone words have been issued by the International Civil Aviation Organization (ICAO) to eliminate the misunderstanding of ATC speech [6], which makes ATC speech significantly different from daily speech. Developing ASR systems that accurately transcribe these domain-specific terms is a substantial challenge.
- Domain specificity: ATC communication involves industry-specific terminology and communication standards that may be absent or limited in ASR model training data. ASR systems typically require domain-specific data and terminology to achieve a high accuracy. The lack of these domain-specific elements can lead to decreased model performance. ATC speech data cannot be added to the general corpus to avoid interfering with training and affecting the use of the general corpus in other fields.
- Accents and variability: Pilots and ATCos come from diverse linguistic backgrounds and may speak with different accents or dialects. ASR systems must account for this variability to ensure accurate recognition, adding complexity to the training and adaptation processes.
- Unstable speech rate: The pace of communication within the ATC environment is characterized by its inherent variability and unpredictability. This variability arises due to the dynamic nature of aviation operations, where ATCos and pilots must adapt their speaking rates to rapidly changing circumstances. It can fluctuate significantly in response to various factors, such as emergencies, traffic density, radio congestion, language and accent differences and experience.
- Noise and interference: ATC communication often occurs in high-noise environments, including aircraft engine noise, radio communications and other sources of interference. These noise and interference factors can adversely affect ASR performance, necessitating specific handling and adaptation.

Several reviews have summarized the application of ASR technology in ATC. An early review presented the challenges and potential applications of ASR in the ATC domain [7]. However, only traditional ASR methods have been analyzed, while modern approaches such as DNN and E2E have yet to be involved. Subsequently, the other two reviews summarized several European projects, which do not include a comprehensive statement of the current status of ASR research [8,9]. In a review published in 2021, the authors described the use of ASR in ATC from the perspective of challenges and solutions without involving too many specific technologies [10]. With the development of deep learning and E2E technologies, the performance of ASR has been dramatically improved, and ATC research institutions have made more exploration and attempts in the ASR domain. A comprehensive overview of the application of ASR in the ATC domain needs to be provided.

This review offers a comprehensive overview of ASR in the context of ATC, intended as a supplementary resource to the existing reviews on the subject. The main contributions are as follows:

- The fundamental models and techniques employed in ASR research within the ATC domain are introduced, including traditional statistical models, DNN-based acoustic and language models and the E2E model.
- A comprehensive analysis is offered from various perspectives, including ATC corpora, models, evaluation measures and applications.
- Recognizing the significance of evaluation measures, we have established a comprehensive evaluation framework. This framework enables a thorough assessment of the developed ASR system, facilitating the identification of shortcomings and the implementation of practical improvements.
- A clear outline of the challenges is provided, along with strategies to enhance the effectiveness of ASR in the ATC domain.

This review is organized as follows. Section 2 introduces the statistical model and E2E model involved in the summarized approaches. Section 3 describes the critical contents of each method, including corpora, models, evaluation measures, and applications. Section 4 presents the evaluation framework for ASR systems in the ATC domain. Section 5 explores the challenges and obstacles in current methods and offers innovative solutions for the future. Section 6 concludes this review.

## 2. Key Models and Technologies for Automatic Speech Recognition

Before 2009, the core component of ASR systems was the statistical models that represented the various phonemes and phonetic features of the target language to be recognized. The Gaussian mixture model (GMM) was employed in conjunction with the hidden Markov model (HMM) to establish a suitable framework for building such models [11]. Advancements in computer hardware and machine learning algorithms have significantly improved the efficiency of training DNNs. With the adoption of novel training methods, DNNs can outperform GMMs in acoustic modeling for ASR, particularly on diverse datasets, including those with extensive vocabularies [3]. Since 2015, continuous improvements in deep learning models, the emergence of E2E models, and the adoption of self-attention mechanisms have led to significant advancements. As a result, ASR systems have become more accurate and adaptable to diverse speech scenarios [4]. The general ASR models and techniques involved in this review are described in this section.

### 2.1. Statistical Model

**Figure 2:**
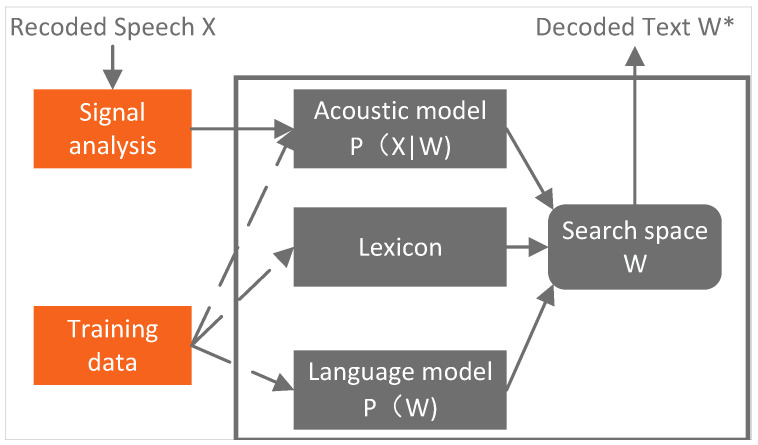
Structure of the statistical model.

A statistical model comprises an acoustic model (AM), a language model (LM), a lexicon, and a decoder, all collaborating to execute ASR tasks. The AM is responsible for transforming sound signals into acoustic feature sequences. Subsequently, the decoder utilizes the output from AMs, along with information from LMs and lexicons, to embark on a search for the most probable text output. Ultimately, the decoder generates the optimal text sequence, constituting the recognized result of ASR. This process represents a dynamic search challenge, wherein the decoder must navigate a vast search space to obtain the most accurate speech recognition outcomes. Hence, ASR systems necessitate efficient algorithms and a substantial amount of training data to attain a high performance. The structure of the statistical model is shown in Figure 2.

With the speech signal being $X=[x_{1},x_{2},x_{3},\cdots ]$ and a word sequence being $W=[w_{1},w_{2},w_{3},\cdots ]$, the target of the ASR task can be described as follows:

(1) $$\begin{array}{cc} W^{*} & =\arg\max_{W}P\left(W|X\right)\\ \; & =\arg\max_{W}\frac{P(X|W)P(W)}{P(X)}\\ \; & =\arg\max_{W}P\left(X|W\right)P\left(W\right) \end{array}$$

where P(X|W) is predicted by the AM, and P(W) is calculated by the LM.

#### 2.1.1. Acoustic Model

The AM is the initial component of an ASR system, which is responsible for analyzing sound signals into acoustic features or vectors. It utilizes pre-trained models to map acoustic features to probability distributions at the level of phonemes, speech units, or subword units. It determines the probability of each time step in the acoustic feature sequence corresponding to a particular speech unit. As noted above, P(X|W) can be expressed in the form of a hidden Markov chain:

(2) $$P\left(X|W\right)=\prod_{l=1}^{L}P\left(x^{w_{l}}|w_{l}\right)$$

where each $x^{w_{l}}$ is a valid pronunciation for the word $w_{l}$, each base phone *x* is represented by a continuous density HMM with transition probability $P(w_{j}|w_{i})$ and emission probability $P(x_{j}|w_{i})$. The transition probability can be calculated from samples using conventional statistical methods, but the main difficulty is calculating HMM emission probability.

HMM establishes the framework for ASR, and the modeling of its emission probability directly impacts the performance of AM. GMM, with a sufficient number of sub-Gaussian components, can effectively approximate a wide range of probability distributions, making it a commonly used choice for the emission probability model. After training the GMM, the emission probability can be computed by comparing each probability density function. Subsequently, P(X|W) can be calculated by combining the initial and transition probability of HMM. Given a trained GMM-HMM model and a speech sequence *X*, it becomes possible to calculate P(X|W) for different alternatives of *W*.

A DNN replaces the GMM to model the emission probability of the input speech signal in a DNN-HMM model. DNN is a deep learning model with multiple hidden layers, allowing it to learn nonlinear feature representations, thereby capturing complex patterns within speech signals more effectively. In the DNN-HMM model, a DNN takes several frames of coefficients as input and generates posterior probabilities over HMM states as output. It is data-driven, enabling it to automatically learn acoustic features and speech patterns from large-scale speech data without manual feature engineering. This flexibility allows DNN to better adapt to variations in speaker pronunciation, speech scenarios and diverse speech backgrounds.

Both convolutional neural networks CNNs and long short-term memory (LSTM) networks have demonstrated significant improvements over DNN in various ASR tasks [12]. CNNs can reduce frequency variations and capture speaker-related features while filtering out input variations. LSTMs are proficient in temporal modeling, making them suitable for modeling sequential information. Combining these networks in a unified framework has the potential to enhance ASR performance by effectively addressing both spectral and temporal aspects of speech recognition.

#### 2.1.2. Language Model

An LM considers the language structure and context of text. The role of an LM is to provide additional information to make text predictions more accurate. It models the probability distribution of word sequences P(W) to determine the most likely combinations of words within a given context. The probability of a word sequence $W=(w_{1},w_{2},\cdots ,w_{n})$ is expressed as follows:

(3) $$\begin{array}{cc} P(W) & =P\left(x_{1}=w_{1},\cdots ,x_{n}=w_{n}\right)=P\left(w_{1}^{n}\right)\\ \; & =P\left(w_{1}\right)P\left(w_{2}|w_{1}\right)P\left(w_{3}|w_{1}^{2}\right)\cdots P\left(w_{n}|w_{1}^{n-1}\right)\\ \; & =\prod_{k=1}^{n}P\left(w_{k}|w_{1}^{k-1}\right) \end{array}$$

where:

(4) $$P\left(w_{k}|w_{1}^{k-1}\right)=\frac{count(w_{1},w_{2},\cdots ,w_{k})}{count(w_{1},w_{2},\cdots ,w_{k-1})}$$

The count() function represents the number of times a word string appears in the corpus. Smoothing algorithms can be used to solve the problem of some word strings not appearing in the training data due to factors such as insufficient training corpus or uncommon word strings. The commonly used LMs are based on N-gram LMs, neural network-based LMs, or pre-trained LMs.

The parameters of the model increase exponentially with the length of the string, making accurate estimation nearly impossible. The Markov assumption was introduced to address this challenge: the probability of a random word appearing is only related to the finite number of N−1 words that appear that precede it. This statistical LM based on the above assumption is called the N-gram LM. Using N-gram, the probability of a word sequence $W=(w_{1},w_{2},\cdots ,w_{n})$ is expressed as follows:

(5) $$\begin{array}{cc} P(W) & =P(x_{1}=w_{1},\cdots ,x_{n}=w_{n})\\ \; & =P\left(w_{1}\right)P\left(w_{2}|w_{1}\right)P\left(w_{3}|w_{1}^{2}\right)\cdots P\left(w_{n}|w_{n-N+1}^{n-1}\right)\\ \; & =\prod_{k=1}^{n}P\left(w_{k}|w_{k-N+1}^{k-1}\right) \end{array}$$

where:

(6) $$P\left(w_{k}|w_{k-N+1}^{k-1}\right)=\frac{count(w_{k-N+1}^{k-1}w_{k})}{count(\left(w_{k-N+1}^{k-1}\right)}$$

From the perspective of model performance, the larger the value of *N*, the better its performance. However, as *N* is increased, the extent of performance improvement decreases.

Neural network-based LMs represent a significant leap forward. Unlike traditional N-gram LMs, these models leverage deep learning techniques such as recurrent neural networks (RNNs) or transformers to comprehensively and intelligently understand and predict text. One key advantage of these neural network-based LMs is their ability to capture context within text sequences beyond considering the current vocabulary item. They excel at predicting the next vocabulary item through effective context modeling, enhancing ASR accuracy.

The task of the LM involves predicting a sequence where each result relies on preceding data. RNN is a model that is used to solve sequence problems, where all previous words will affect the prediction of the present word. Therefore, using RNN to predict the current word using historical vocabulary in sentences, LM can be constructed. RNN has a broad output layer, which covers the vocabulary LM uses. The output of each node represents the probability of the generated word $P(w_{k}|w_{1},w_{2},\cdots ,w_{k-1})$, and P(W) can be calculated as follows:

(7) $$P\left(W\right)=\prod_{k=1}^{n}P\left(w_{k}|w_{1},w_{2},\cdots ,w_{k-1}\right)$$

The RNN LM can use the same neural network to process any length of historical information, while the size of the N-gram LM exponentially increases as N increases. The N-gram LM stores the possibilities of various vocabulary combinations, which can be directly edited, such as N-gram LM fusion of different domains, new word addition, etc. However, parameters in the RNN LM cannot be modified, making it challenging to expand new words. When using RNN LM, $P(w_{k}|w_{1},w_{2},\cdots ,w_{k-1})$ is temporarily calculated, which leads to low real-time performance. On the contrary, $P(w_{k}|w_{k-N+1}^{k-1})$ is directly stored in the N-gram LM, which saves decoding time.

Pre-trained LMs are a type of LM that has recently emerged, with bidirectional encoder representations from transformers (BERT) [13] being a prominent example. These models are trained on extensive text data to learn contextual information from the text. They can be fine-tuned for specific tasks like ASR. They often perform exceptionally well because they can grasp the deep semantic structures of the text.

#### 2.1.3. Lexicon

In ASR, a lexicon is a table that maps words to pronunciations. It contains a vocabulary list encompassing all possible words that the ASR system needs to recognize, along with their pronunciation information. Additionally, the lexicon may include other linguistic features of words, such as stress patterns, parts of speech, variants, etc., to aid the system in better understanding and transcribing the speech signals accurately.

Using a lexicon, the mapping relationship between AM modeling units and LM modeling units is obtained, which connects AM and LM to form a search space for decoding by the decoder. A precise and comprehensive lexicon can significantly enhance the system’s recognition rate and adaptability, especially when dealing with various speech signals, accents, language variations, and application scenarios.

#### 2.1.4. Decoder

The ASR decoder is a critical component that is responsible for transforming acoustic feature sequences into textual output. Its primary function is to find the optimal transcription result using a search algorithm, given the acoustic input. The objective of the search algorithm is to identify the word sequence with the highest probability, considering the combined influence of the AM (typically representing phoneme probability distributions), the LM (which models language structure and context in the text), and the lexicon. This search for the highest-probability word sequence is often accomplished using algorithms such as the Viterbi algorithm. These algorithms leverage the collaborative interaction of the AM, LM and lexicon to determine the most likely transcription of the acoustic input.

The function of the decoder is to select a *W* that maximizes P(W|X); that is, to search for the optimal path in the search space. The Viterbi algorithm is widely employed for HMM-based ASR systems. It efficiently finds the most likely sequence of hidden states given the observed acoustic features [14]. Beam search is an optimization of the Viterbi algorithm, where a beam width parameter limits the number of candidate paths considered during decoding. This reduces the computational complexity while maintaining a good performance. The breadth-first search is used in the dynamic decoder to generate multiple hypotheses simultaneously in the original search network, and it relies on pruning algorithms to prevent the network from becoming too large. A static network is constructed and called directly by extracting and compiling dynamic knowledge sources, which can accelerate decoding speed.

### 2.2. End-to-End Model

Traditional statistical ASR models have made significant advances in various fields but still exhibit several critical limitations. Firstly, the statistical ASR systems involve multiple components, including AMs, LMs, lexicons, and decoders, making system development and maintenance cumbersome. Additionally, building these models requires the involvement of domain experts who manually design acoustic and language models, limiting the system’s applicability and flexibility. Furthermore, traditional ASR methods are highly demanded for large-scale annotated speech data, which are costly and time-consuming to acquire and label. Moreover, acoustic feature engineering is a necessary step in traditional ASR methods, which entails converting audio signals into feature representations that are suitable for model processing, potentially leading to information loss. Additionally, traditional ASR methods typically rely on external language models and phonetic dictionaries, which may not be suitable for specific application scenarios. Finally, these methods are sensitive to noise and speaker variations, which can result in decreased performance in real-world applications.

**Figure 3:**
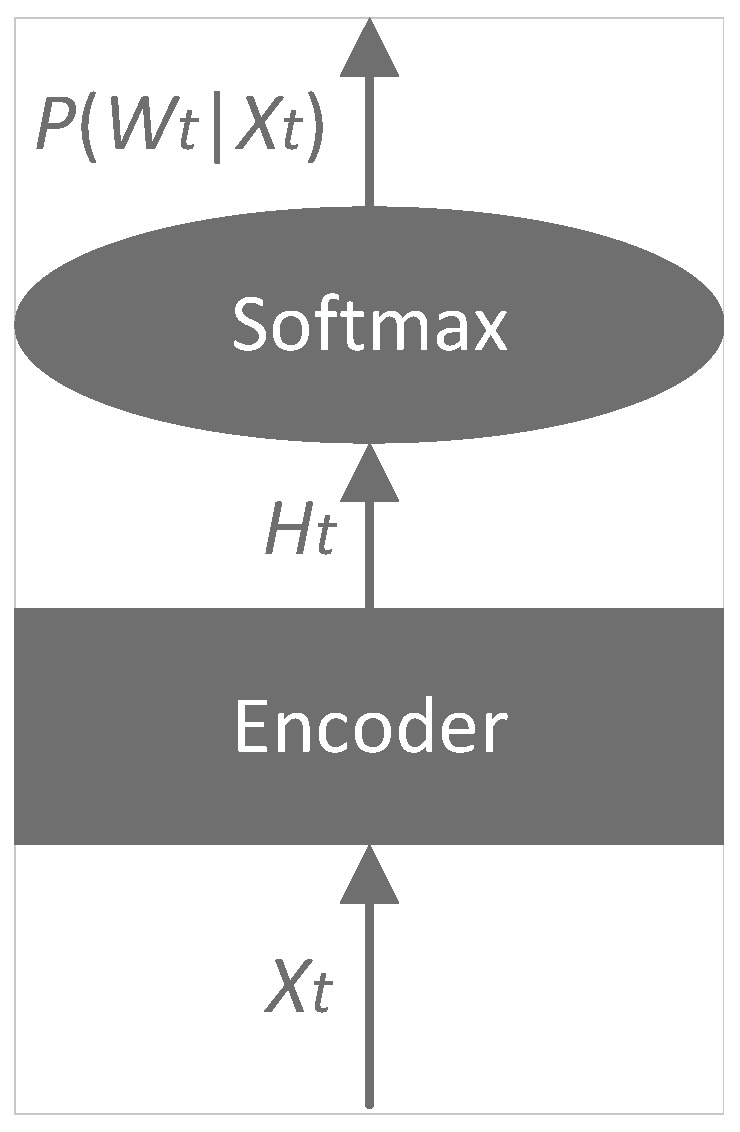
The architecture of connectionist temporal classification.

Modern ASR research has adopted E2E approaches to overcome these limitations, which directly map audio signals to text, bypassing multiple intermediate steps. The E2E models simplify the ASR pipeline by optimizing the entire network with a single objective function aligned with the ASR objective [4]. Some of the most commonly used E2E techniques for ASR include: (1) connectionist temporal classification (CTC) [15], (2) attention-based encoder–decoders (AEDs) [16] and (3) recurrent neural network transducers (RNN-T) [17]. This section briefly describes CTC, which is used for ASR in the ATC domain. Figure 3 shows the architecture of CTC.

The CTC technique for ASR was designed to create CTC paths that map the input speech sequences into output label sequences. The input speech sequence is denoted as *X*, while the original output label sequence is represented by *W*, and all CTC paths that are mapped from *W* are denoted as $B^{-1}\left(W\right)$. The encoder network transforms the acoustic feature $X_{t}$ into a sophisticated representation $H_{t}$. The CTC loss function is utilized to determine the negative logarithmic probability of the correct labeling of speech sequences based on the input data:

(8) $$L_{CTC}=-lnP\left(W|X\right)$$

with:

(9) $$P\left(W|X\right)=\sum_{Q\in B^{-1}\left(W\right)}P\left(Q|X\right)$$

where *Q* is a CTC path. With the conditional independence assumption, the expression P(Q|X) can be broken down into a series of frame posteriors as follows:

(10) $$P\left(Q|X\right)=\prod_{n=1}^{N}P\left(q_{n}|X\right)$$

where *N* is the length of the speech sequence. The encoder in an E2E model is a critical component that transforms input speech sequences into feature representations at a high level. The typically used encoder is a multi-layer bidirectional LSTM (BiLSTM).

## 3. Application

### 3.1. Overview

The utilization of ASR in the ATC domain aims to enhance flight safety, increase ATC efficiency, and reduce the workload of ATCos. This section summarizes the findings of nearly all published papers in recent years, as presented in Table 1.

Researchers have long recognized the benefits of using ASR in the ATC domain and have conducted exploratory research. The ATCOSIM project, funded by Eurocontrol, assessed the need for ASR corpora and constructed a professional ATC corpus [18]. A corpus comprising both the English and Spanish languages has been developed to cover all stages of tower control, and the ASR was performed using a combination of HMM-based AM and parallel phone recognition (PPR)-based LM [19,20,21]. CRIDA implemented ASR for callsign recognition, aiming to reduce the workload of ATCos [22,23]. In the AcListant project, the ASR technology was utilized to aid the arrival manager (AMAN) and ATCos by reducing manual data input requirements [24,25,26,27,28,29,30]. As part of this project, an ATC corpus was created, incorporating accents from German and Czech speakers. Furthermore, the project has succeeded in reducing ATCos’ workloads. The FAA in the United States also conducted ASR research to check for incorrect instructions issued by ATCOs [31]. Nguyen et al. proposed a method to reduce word error rates (WER) using semantic understanding [32]. Optimal Synthesis presented the concept of the pseudo-pilot, used a comprehensive ASR model and evaluated the workload of ATCos [33].

With advanced deep learning technology, DNNs have also been widely used in ASR research in the ATC domain. The ATCAIR-BUS corpus was built by Airbus, and the ASR Challenge was organized based on this corpus, resulting in various advanced models in 2018 [34,35,36]. Additionally, the Civil Aviation University of China conducted a study on repetition checking, but only matched through templates without utilizing ASR technology [37]. They also leveraged DNN-HMM to enhance ASR performance in the ATC domain [38]. The MALORCA project was oriented towards leveraging machine learning algorithms to create a cost-effective and adaptable approach for assistant-based speech recognition (ABSR) in specific environments [39,40,41,42,43,44]. The ATC corpus of the Prague and Vienna airports has been constructed based on this project. Additionally, various training methods based on deep learning have been proposed. The CWI HMI project aims to increase the productivity of ATCos and support ASR industrialization in the ATC domain [45,46,47]. Research in this stage encompasses a range of innovative ideas, including reducing the speed of speech to improve the recognition accuracy [48], combining with commercial software [49] and constructing a comprehensive corpus [50,51], among others.

With the development of E2E models and the growing maturity of DNNs, many advanced ASR studies have emerged in the ATC domain since 2019. These studies focused on constructing a comprehensive corpus, improving recognition performance, and practical applications. The Nanjing University of Aeronautics and Astronautics used an E2E model that combines CNN, BiLSTM and CTC [52]. Sichuan University made outstanding contributions in corpora, models, algorithms and applications [53,54,55,56,57,58,59,60,61,62,63]. They built an ATC corpus called ATCSpeech that includes Chinese utterances and English utterances with Chinese accents, and it is a multilingual corpus [56]. A hybrid E2E model [54,55,59,60,61,62] and a cascaded [53,57,58] model were proposed, significantly improving ASR performance. In addition, the ASR system was applied and continuously improved in the operation department of ATC [10]. MIT also designed an advanced model and validated it using different corpora [64]. The ATCO2 project mainly focused on constructing a corpus, providing a detailed description of the construction methods and data requirements for the corpus [65,66,72,73,74,75,76,77,78,79,80,81]. The HAAWAII project, on the other hand, leaned towards application, focusing on improving safety and reducing the workload of ATCos [65,67,68,69,70,71,72,73,78,79,80,81].

This section delves into the essential components of ASR in the ATC domain. The analysis is based on paper research and highlights the importance of corpus construction, models and their extensions, evaluation measures and application scenarios.

### 3.2. ATC Corpora

The corpus serves as the cornerstone for implementing ASR and sets the ATC corpus apart significantly from general corpora. Collecting and annotating data in ATC communication voices poses formidable challenges due to the inherent characteristics of noise, unstable speech rates, and code-switching. Early ATC corpora varied widely in scope, technical conditions and accessibility to the public. The ATCOSIM corpus aims to bridge the gaps left by these earlier corpora. In the realm of ASR research within the ATC domain, a range of outstanding ATC corpora, such as AcListant, AIRBUS-ATC, MALORCA, HAAWAII, ATCO2, ATCSpeech and others, were meticulously constructed. Table 2 in this article provides a summary of the ATC corpora discussed.

Early ASR research in the ATC domain predominantly depended on general corpora, with minimal emphasis on ATC-specific corpora. Consequently, ASR performance in the ATC domain has consistently fallen short of expectations. The Air Traffic Control Complete Corpus (LDC94S14A) [82] was produced in 1994, composed of recordings of 70 h of utterances at three airports in the US. The database is formatted according to NIST Sphere standards and contains complete transcripts as well as precise start and end times for each transmission. It is a valuable resource for speech recognition research, but it has limitations such as limited diversity and a relatively small dataset size. The NATO research group has created the NATO Native and Non-Native (N4) Corpus [83], which encompasses multiple languages and accents from NATO member countries to represent the diversity of international military communications. Its advantages include extensive coverage across various languages, offering a rich resource for multilingual ASR research. However, its drawbacks may include high costs associated with data collection and maintenance, as well as potential security and access restrictions due to its involvement in military communications. The HIWIRE corpus [84] comprises 8,099 studio-recorded utterances spoken by non-native speakers, with artificially added cockpit noise. Another corpus includes 7.1 h of Spanish and 4.7 h of English utterances, encompassing various aviation scenarios at the Madrid airport, such as clearances, takeoffs, arrivals and taxiing [21]. Additionally, the Madrid ACC corpus, totalling 100 h of utterances, covers en-route and approach communications during regular operations at the Madrid Area Control Center [22,23].

The ATCOSIM corpus [18] aims to provide authentic ATC speech, accurately representing speaking style, languages, noise and stress levels across various situations. This extensive data repository comprises 10 h of utterances information recorded during authentic ATC simulations utilizing a close-talk headset microphone. The utterances are pronounced by ten non-native speakers in English. Remarkably, it stands as the inaugural civil aviation ATC corpus that is freely available for download, serving as a foundational resource for training and testing purposes in numerous subsequent studies.

In the AcListant project, a corpus was created through a standard process that involves simulation, transcription and annotation [25]. The corpus consists of recordings of three ATCos—a male German, a female German and a male Czech native speaker—issued in English. The ATCSC corpus includes 4800 clearances generated using ICAO standardized phraseologies [32].

The AIRBUS-ATC corpus [34] considers the specific features of ATC, including non-native speech, poor audio quality, code-switching and rapid speech rates, which is designed to develop an ASR system that is capable of processing ATC communications. In the Airbus Air Traffic Control Speech Recognition 2018 Challenge, the corpus was partitioned into three datasets for training, testing and validation [35]. In recent years, this corpus has also become a commonly used benchmark dataset in various research studies.

The Prague and Vienna approach datasets were collected as part of the MALORCA project [39]. Both datasets contain ATCos speech only and have a high signal-to-noise ratio (SNR), making them high-quality corpora. Transcribing and annotating ATC communication voices demand substantial personnel involvement and time resources. A noteworthy contribution of this project lies in the utilization of a semi-supervised learning algorithm that effectively incorporates numerous unlabeled data to complement the limited labeled data.

UWB-ATCC [50,51] is a publicly available corpus of ATC communication recordings between ATCos and pilots, which was recorded at the Air Navigation Services of the Czech Republic and manually annotated. It is designed to assist researchers in developing and evaluating UWB-ATCC system components, including speech recognition, communication quality and overall performance, to meet the high demands of flight safety and control efficiency. It includes separate sets for training, development and testing.

SOL-Twr [67] and SOL-Cnt [76,80] were recorded and collected during SESAR-2020-funded industrial research projects. The ATC communication voice in SOL-Twr originated from the Lithuanian Air Navigation Service Provider (ANSP), whereas the ATC communication voice in SOL-Cnt was sourced from the Vienna approach. The SOL-Twr voice recordings exhibit lower noise levels than SOL-Cnt due to the controlled laboratory recording environment.

The corpus used in the HAAWAII project was collected and annotated by the London approach (NATS) and Icelandic en-route (ISAVIA) [76]. Approximately 19 h of voice recordings were manually transcribed and annotated for the NATS portion, while the ISAVIA segment offers 15 h of similarly transcribed and annotated voice data [78].

Two corpora were constructed in the ATCO2 project. The ATCO2 test set corpus was built to develop and evaluate ASR technology for English ATC communications. The data in this corpus was transcribed and annotated, with 1.1 h being free and the other 3 h requiring purchase. The ATCO2-PL-set corpus represents a substantial and notable collection, comprising an extensive repository of over 5281 h of ATC utterances originating from 10 different international airports. This corpus stands as a significant achievement, representing the largest and most comprehensive ATC dataset currently available. Notably, the data within this corpus were automatically transcribed without the inclusion of manual annotations.

ATCSpeech is a specialized data collection that trains accurate ASR systems specifically for ATC purposes [56]. It is the only publicly available corpus for the ATC application with accented Mandarin Chinese and English utterances. It includes 39 h of Chinese and 19 h of English utterances, covering the ground, tower, approach and en-route phases.

LiveATC [85] is an online platform dedicated to providing real-time recordings of ATC communications. It depends on the generous contributions of community members residing in close proximity to airports, who graciously lend their VHF receivers to capture and archive stream recordings. This coverage spans various types of control, including tower control, ground control, approach control and more. Although this dataset contains raw speech data that have not been transcribed or annotated, available data can be selected to supplement the corpus due to its comprehensiveness and richness. On the one hand, it can be manually transcribed and labeled. On the other hand, semi-supervised or unsupervised learning algorithms can be used to utilize unlabeled data.

There have been many achievements in corpus construction. In accordance with ICAO regulations, English serves as the prescribed language for ground–air communication in international flights, while native languages are permissible for domestic flights. These constructed corpora typically encompass both standard English and English with various accents. A select few also incorporate native languages such as Chinese and Spanish, ensuring diversity. These corpora cover all four phases of ATC, including ground, tower, approach and en-route. Speech data can be obtained through actual operation or simulation. A corpus constructed using actual data meets the speech rate, noise and code-switching demands, but its acquisition is difficult. Simulation data are generally obtained in a laboratory environment, with good speech quality, but they do not conform to the actual operation of ATC. The availability of these corpora varies, with some being freely accessible and others requiring payment. Researchers can select specific corpora based on their study objectives or employ multiple datasets for comprehensive training, testing and validation. Additionally, LiveATC offers a unique opportunity where speech recordings can be downloaded, transcribed and annotated, serving as a supplementary resource to enrich existing corpora.

The researcher can construct the ATC corpus themselves [33,37,38,52]. To ensure the quality and relevance of the corpus, data collection should align closely with the study’s specific scope, avoiding the inclusion of excessive data with limited value. Subsequently, the collected data must undergo transcription and annotation processes. Transcription involves the systematic conversion of spoken words into written form, serving as a fundamental step in corpus creation. Annotation, following transcription, takes this a step further by associating spoken words with pertinent ATC concepts, enhancing clarity and comprehensibility. It goes beyond mere textual representation of utterances and includes additional context, such as identifying segments corresponding to callsigns, command types, command values and more. A data augmentation technique can be implemented to counteract issues within the training sets [76]. In the case of insufficient labeled data, a semi-supervised learning method can use unlabeled data effectively.

### 3.3. Models and Their Extensions

A model tailored to the specific problem is necessary to utilize an ASR system effectively. It can be achieved through various options, including traditional HMM models, E2E models and cascaded/hybrid models. The appropriate model selection will depend on the nature of the problem being addressed and the application’s specific requirements. The ASR models used in the ATC domain consist of feature extraction, acoustic models, language models and decoders. The models utilized in the research articles featured in this review are summarized in Table 3.

#### 3.3.1. Feature Extraction

Feature extraction plays a crucial role in ASR within the context of ATC. The feature extractor converts analogue speech signals into acoustic feature vectors, commonly using filter banks (FBanks) or Mel frequency cepstrum coefficients (MFCCs) to reduce training and decoding time [86].

The MFCC feature extraction method transforms the raw audio waveform into a series of feature vectors that contain spectral information. This is achieved through several steps, including segmenting the audio signal into frames and applying a short-time Fourier transform (STFT). MFCC features are then computed from these frames and used as inputs to train ASR models. However, this approach may result in the loss of some fundamental speech information, as it is based on the human ear’s response to audio [36].

Cepstral mean and variance normalization (CMVN) is a common technique used to normalize the MFCC features to make them consistent for ASR tasks [19,20,21]. Cepstral mean normalization involves calculating the mean of the MFCC coefficients over a specified context window. This mean is then subtracted from each frame’s MFCC coefficients. Cepstral variance normalization, on the other hand, calculates the variance in the MFCC coefficients over a context window. This variance is then used to scale the MFCC coefficients for each frame. The MFCC processed using the CMVN technique can be utilized to normalize the features at the utterance level’s mean and variance statistics while training the mono-phoneme model [38]. Applying CMVN to MFCC features enhances their robustness against variations in speech recordings, environmental conditions and speaker characteristics.

CNNs can be used to extract learnable features from raw waveforms, supporting acoustic modeling in ASR. A novel feature learning block was proposed to extract informative features from raw waveforms. It involved concatenating feature maps derived from multiple learning paths, including SincNet and conventional convolutional paths [54]. A hybrid speech feature was innovatively developed for utilization as input in E2E ASR models to tackle challenges related to suboptimal speech quality and limited feature distribution. This approach effectively enables the model to learn underlying patterns from diverse feature engineering types. The hybrid feature-embedding block utilized CNN layers to establish spatial correlations and reduce data size [62]. These advancements in feature extraction through CNNs and hybrid feature embeddings contribute significantly to the evolution of ASR systems in the ATC domain, enabling them to adapt to diverse speech conditions and yield improved performance.

#### 3.3.2. Acoustic Model

The utilization of AMs in ASR research within the ATC domain aligns with the broader developmental trajectory of ASR technology. In the nascent phases of ASR research, the conventional GMM-HMM model was predominantly employed. However, during this period, there was a notable absence of significant innovations or enhancements to the model. As deep learning gained prominence and reshaped the landscape of ASR, the DNN-HMM model also found its application in the ATC domain. Nonetheless, the DNN model primarily adopted a fully connected (FC) structure without incorporating novel architectural designs or structures.

CNN and LSTM are two significant categories of DNNs and have been widely used in ASR research in recent years. These two types of neural networks can be employed independently, such as using LSTM [36,64] alone or using CNN alone [38]. However, the most advanced research projects, including those conducted by Sichuan University, the HAAWAII project and the ATCO2 project, utilize a combination of models. Due to the development of CTC attention, in recent hot research on E2E models, an FC is also used to connect CTC after the CNN and LSTM layers. CTC serves not only as part of the AM but also operates as a decoder.

**Figure 4:**
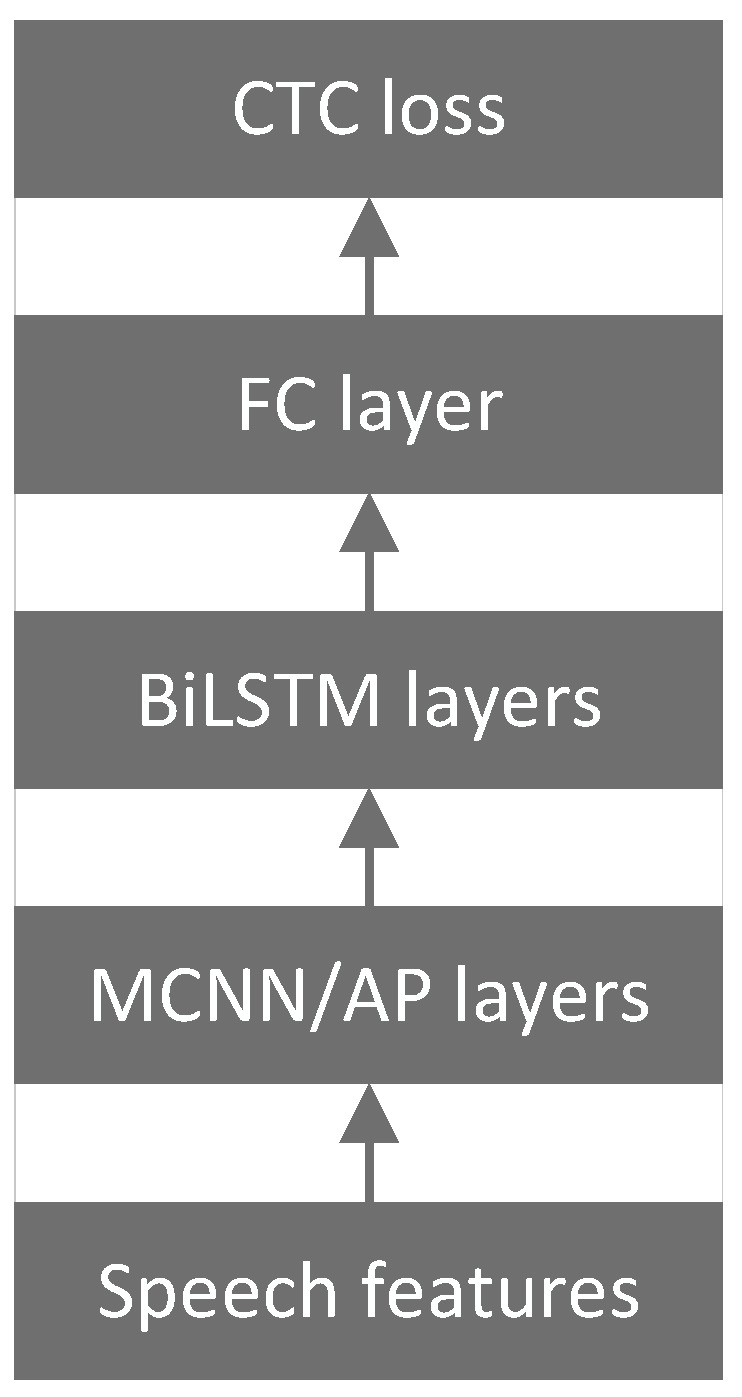
Acoustic model in the cascade model and E2E model.

Sichuan University developed two distinct ASR architectures: the cascade model [53,57] and the E2E model [54,55,56,58,59,60,61,62]. Both the cascade model and the E2E model employed an identical AM, comprising a CNN, BiLSTM layers and CTC, as illustrated in Figure 4. An architecture utilizing a multiscale CNN (MCNN) and average pooling (AP) was designed to address specific challenges encountered in ASR in the ATC domain. This architecture effectively leverages spatial correlations within spectrograms and adeptly handles complex background noise and high speech rates. The MCNN emphasizes the construction of various resolutions along the frequency dimension while simultaneously modeling correlations along the temporal dimension. The AP operation selectively filters out high-intensity noise, enhancing the clarity of human speech and facilitating the extraction of discriminative features that are crucial for subsequent sequential modeling. Temporal dependencies among speech frames were analyzed through the incorporation of BiLSTM layers within the MCNN/AP architecture, accounting for their temporal characteristics. The CTC loss function was then employed to quantify the disparity between predicted outputs and actual labels, facilitating the training of the neural network.

A novel approach to address multilingual ASR challenges was introduced by the cascade model, which consolidated multiple components into a single unified model. It included an AM, a pronunciation model (PM) and phoneme- and word-based LMs. Given the linguistic diversity encountered at international airports and routes, where ATCos communicate in Chinese for domestic flights and English for international flights, this cascading model effectively tackles multilingual ASR problems in a streamlined manner.

**Figure 5:**
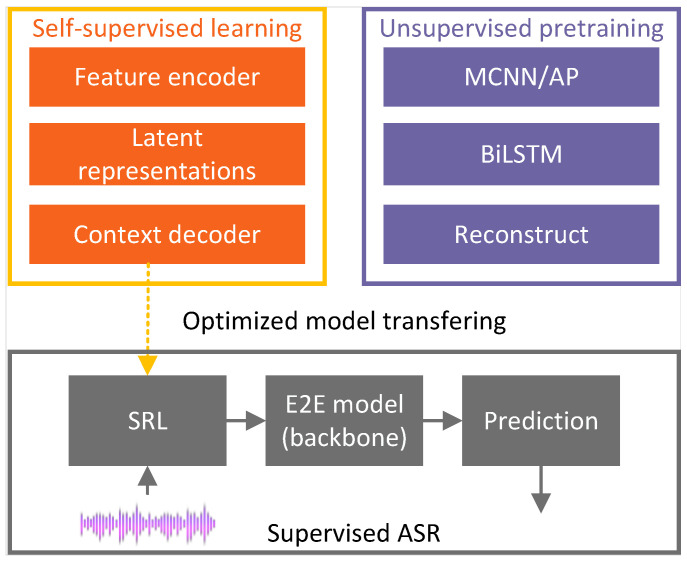
The improved E2E model.

A standard E2E mode, which solely relies on an AM composed of a CNN, an LSTM and a CTC, was initially proposed [54]. However, this model’s performance was found to be less than satisfactory. To enhance its capabilities, an improvement was introduced by incorporating an RNN-trained LM after the E2E model for better performance [55]. However, due to the use of an LM, it is not strictly an E2E model. In their subsequent research, a complex framework was proposed to augment the E2E model’s performance, as shown in Figure 5. This comprehensive approach centers around a CTC-based E2E model, serving as the backbone network. Developing a speech representation learning (SRL) model is a crucial focus aimed at capturing robust and discriminative features from unlabeled audio data through a wave-to-feature paradigm. This approach involved applying self-supervised learning to optimize the model, resulting in a highly effective solution. A solution was introduced to tackle the issue of limited labelled ASR data in the ATC domain, involving an unsupervised pretraining strategy. This strategy optimized the backbone network through feature-to-feature training using unlabeled audio data. Furthermore, optimizing the ASR model incorporated transfer learning for subdomain adaptation through supervised learning. These combined efforts aim to deliver a practical and reliable ASR system that fulfills the specific requirements of the ATC domain.

In the ATCO2 and HAAWAII projects, the time delay neural network (TDNN) [87] framework was mainly used to construct an AM, including HMM-based TDNNF and CNN-based TDNNF [88]. TDNN is a feedforward neural network that processes sequential data by applying different weights to input features at various time steps, capturing temporal dependencies in the data. It involves a modular and incremental design that enables the creation of more extensive networks by combining sub-components. TDNNF is a compressed TDNN with semi-orthogonal matrix constraints that is trained randomly, providing substantial improvements over TDNN.

While enhancing the AM can effectively address speech rate and noise issues in the ATC domain, it does not directly tackle the core challenges associated with code-switching and domain-specific characteristics. Consequently, most efforts in this domain have focused on fine-tuning the AM’s neural network structure or incorporating auxiliary modules. More improvements are being made to the LM for ASR in the ATC domain.

#### 3.3.3. Language Model

The LM incorporates the specific vocabulary and phraseology used in ATC communication, following standard syntax and grammar structures. It also includes logical relationships, probabilistic modeling and language restrictions, rendering it an indispensable tool for ASR in the ATC domain.

In the early stage, grammar, which defines the language rules and structure of the ATC commands, was mainly used as the LM. It was designed to ensure that the generated text or speech adheres to proper syntax, semantics and grammatical constraints. In the context of ASR, a grammar LM plays a crucial role in enhancing the accuracy and fluency of transcribed or recognized speech by favoring grammatically correct sequences of words and phrases.

An innovative approach involving a stochastic bigram was employed to overcome the limitations of traditional rigid grammar files. This bigram covered the standard predefined protocol sentences and accounted for nuanced and individual syntactic variations [20,21]. The AcListan project utilized grammar as a decoding graph component that was consistently updated with context-dependent versions in ASR [26].

ATC data showed that more than one-fourth of the issued commands do not follow the standard ICAO phraseology, making it difficult to create proper grammar-based LMs and substantially decreasing ASR performance. Consequently, N-gram models emerged as the predominant choice for ASR in the ATC domain and have been widely adopted in various studies. They predict the next word based on the frequency of vocabulary sequences observed in previous textual data, helping capture common phrases and usages in ATC communication. They enhance the ASR system’s understanding of specific terms and phrases, thereby improving recognition accuracy. However, due to factors like limited training data or infrequent word sequences, certain word combinations may not be well-represented in the training text. Different smoothing algorithms, such as interpolation and back-off, can be applied to address this.

One effective method for establishing contextual relationships is through the use of a neural network, which is capable of modeling nonlinear features with excellent proficiency. In particular, an RNN is a common architectural choice for language modeling due to its ability to account for sequential correlations between various vocabulary words. Consequently, the LM trained by RNN was used in some studies and achieved a good performance [36,55,57]. Additionally, an LSTM-based LM was also proposed for the ATC task’s particular word pronunciation and grammatical features [52].

**Figure 6:**
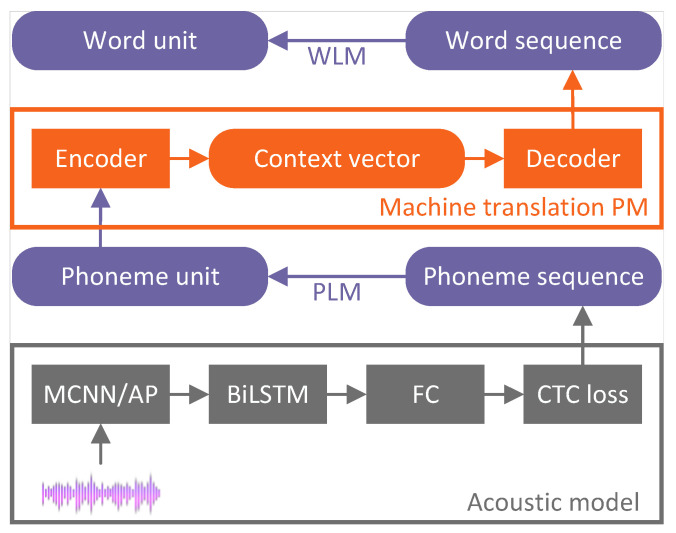
Procedure for the cascade model.

A cascade model proposed by Sichuan University includes sub-models for an AM, phoneme-based LM (PLM), PM and word-based LM (WLM) [53], as shown in Figure 6. The PLM and WLM were specifically designed to facilitate and enhance ASR decoding, with a primary focus on effectively handling code-switching words. These models are then utilized to refine and adjust the AM and PM output under the ATC application’s unique characteristics. The input of PLM is a phoneme sequence, and its output is a phoneme unit, while the input of WLM is a word sequence, and its output is a word unit.

The primary role of the PM is to convert the phoneme sequence generated by the AM or the phoneme unit transformed by the PLM into a sequence based on words, which accurately represents human understanding, enhancing the system’s overall effectiveness. The machine translation PM (MTPM) was built on the concept that phonemes and words are representations of spoken language. It involved mapping a variable-length phoneme sequence to an equivalent variable-length word sequence.

**Figure 7:**
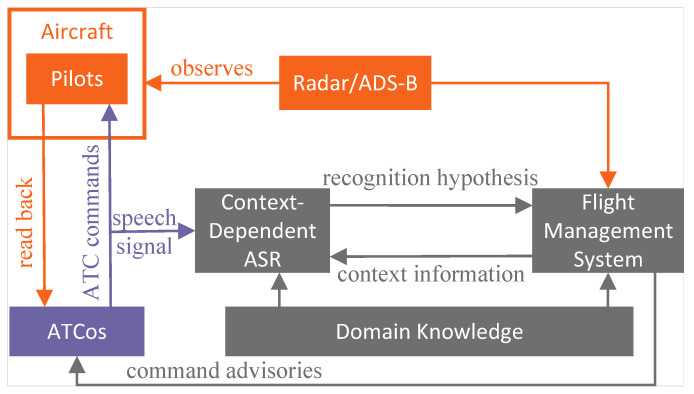
Context information and domain knowledge used in ASR.

In addition to LM, context information and domain knowledge can be used to improve ASR performance, as shown in Figure 7. Context information and domain knowledge are essential factors that enhance ASR performance within the ATC domain. The ATC system is intricately connected to surveillance systems like ADS-B or radar, which provide essential data about aircrafts, airports, and flight routes. By establishing a connection between the surveillance system and ASR and cross-referencing contextual information with the ASR recognition text, errors can be detected and corrected, improving ASR accuracy. Domain knowledge is based on a given environment, which includes the runway names, handover frequency values, waypoint names and coordinates, pronunciations, etc. [43]. These need to be manually added to the lexicon, together with its possible pronunciations, to improve the performance of the ASR system in specific ATC regions or scenarios. Strictly speaking, context information and domain knowledge are not part of ASR but can enhance the performance of ASR in the ATC domain.

#### 3.3.4. Decoder

The decoder is a component that is responsible for translating the model’s output into text or commands. The primary function of the decoder is to translate audio signals into understandable language for communication with pilots or recording purposes. The decoders used in the ASR in the ATC domain include the weighted finite-state transducer (WFST) [89] and CTC decoders.

WFST is typically used in traditional ASR systems, and it is a directed graph data structure used to represent AMs, LMs and the decoding process. It can handle the transcription and decoding of audio signals, allowing for the incorporation of LM information, thus improving recognition accuracy. It is suitable for tasks that require consideration of context and LMs. The decoding graph for WFST is constructed from the four essential components: *H*, *C*, *L* and *G*. The components designated as *H* and *C* primarily focus on the phone-level representation of speech. Specifically, the transducer *H* facilitates the conversion of HMMs to GMMs. Meanwhile, transducer *C* plays a crucial role in introducing phone context dependency by transforming context-independent phones into context-dependent ones. The *L* component serves as the pronunciation lexicon within the decoding graph, facilitating the translation of phone sequences into their corresponding words. Ultimately, the *G* component features a WFST representation of the grammar or LM utilized for recognition. The *H*, *C*, *L* and *G* components are integrated to construct the decoding graph for recognition. The equation used in constructing the final decoding graph is as follows:

(11) HCLG=det(H∘min(det(C∘min(det(L∘G)))))

CTC in the E2E model is divided into two components: label alignment and loss function calculation during training, and generating the final prediction during decoding. Following the computation of output results by the AM, the CTC decoder comes into play for decoding purposes, employing either a greedy search or beam search decoding strategy. Greedy search stands out as the simplest decoding method within the realm of CTC decoding algorithms. It involves selecting the best option at each time step, where the symbol with the highest probability in each time step is chosen as the final result during decoding.

The drawback of greedy search decoding lies in its straightforward approach of selecting the element with the highest probability at each time step, resulting in an output composed solely of characters with the highest individual probabilities. This method lacks the ability to fully harness contextual information and make context-aware selections for the most suitable output character. The improved CTC decoder uses a beam search algorithm, which accumulates the probabilities of all character occurrences in the final decoding result and sorts them according to the size of the probability values to provide users with multiple choices.

#### 3.3.5. Training Algorithm

Training algorithms are adopted to train the ASR model using datasets to maximize model performance. Supervised learning is one of the fundamental training algorithms that are commonly used for training the AM, relying on labeled training data. However, when labeled data are limited, unsupervised learning and semi-supervised learning become valuable options, with the former allowing for feature extraction or language modeling from unlabeled data, and the latter combining labeled and unlabeled data. Transfer learning enables knowledge transfer from one domain to another related domain, boosting performance. Self-supervised learning involves learning by maximizing the self-consistency of input data and enhancing feature representations. Reinforcement learning is utilized to train decoders to maximize recognition accuracy. Multi-task learning allows for the simultaneous training of multiple related tasks, which can be leveraged to improve ASR performance. The choice of these algorithms depends on task requirements, available data, and model architectures, often requiring balance and experimentation between different algorithms and strategies to find the most suitable training approach for a specific ASR task.

In the early stage, ASR research in the ATC domain focused on building corpora, with less attention devoted to the development of pioneering training algorithms. In the follow-up research, training algorithms have been enhanced to address the challenges encountered through semi-supervised learning, transfer learning, lattice-free maximum mutual information (LF-MMI), and a combination of different algorithms.

The supervised learning process is costly due to the need for labeled data, while unsupervised learning methods may not consistently yield optimal outcomes. A viable solution is to utilize semi-supervised learning, which offers a beneficial compromise [41,42,44,50,58,74]. Semi-supervised learning aims to leverage large amounts of unannotated data to enhance the performance of the ASR models trained using supervised methods.

Through the utilization of pre-training techniques and the sharing of parameters in a transferred model, the ASR model is able to effectively capture both the data patterns previously learned from the baseline model’s corpus as well as speech representations from a specific unlabeled speech corpus. The transfer learning technique is accomplished via a comprehensive process of supervised learning, wherein the entire model is trained on a unified corpus formed by combining the training data from the baseline model with newly transcribed samples acquired from the target dataset [58,65].

The CTC loss function is instrumental in training E2E models and enhancing ASR performance by automatically aligning speech and label sequences, even when their lengths differ. This loss function quantifies the disparity between predictions and actual labels, facilitating backpropagation to optimize the model’s preceding layers. Several techniques, such as L2 regularization, Gaussian weight noise and frame skipping, have been shown to improve CTC attention performance [52]. The TDNNF-based AM is trained using the LF-MMI approach [90], which involves implementing discriminative sequence training for neural network AMs. This approach eliminates the requirement for pre-training with frame-level cross-entropy.

### 3.4. Evaluation Measures

Evaluation measures have a significant impact on assessing ASR performance within the ATC domain, serving a dual function. Firstly, they gauge the effectiveness of ASR systems in this specialized field. Secondly, they offer valuable insights, highlighting specific areas for enhancement for ASR developers. The current evaluation measures for ASR in the ATC domain mainly contain general measures, including word error rate (WER)/character error rate (CER); sentence error rate (SER); F1 score; real-time factor (RTF); ATC key information measures, including concept error rate (ConER); command error rate (CmdER), callsign accuracy (CSA); and application measures for the ATC domain, including repetition intention accuracy (RIA), acceptable detection rate and workload measurements. The evaluation measures summarized in this article are shown in Table 4.

General ASR evaluation measures include WER/CER, SER, F1-score and RTF. The WER, which measures the difference between predicted and actual labels, is a standard metric for ASR applications, as shown below:

(12) $$WER=\frac{W_{i}+W_{d}+W_{s}}{W}\times 100\%$$

where $W_{i}$, $W_{d}$, $W_{s}$ and *W* denote the replaced, deleted, inserted and total number of words, respectively. The CER, similar to WER, stands as a prevalent metric for evaluating the precision of ASR systems when it comes to recognizing spoken characters. However, due to variations in corpora utilized by different ASR systems or even when using the same corpus with different test sets, it is challenging to measure system performance by comparing WERs in various studies.

*SER* is a metric that is used to evaluate the performance of ASR in recognizing entire sentences. It quantifies the error rate at the sentence level, measuring how accurately the system recognizes sentences. It measures the rate of sentences containing at least one error, as shown below:

(13) $$SER=\frac{S_{e}}{S}\times 100\%$$

where $S_{e}$ denotes the number of incorrectly recognized sentences, and *S* denotes the total number of sentences. A lower *SER* indicates a better performance, as the system correctly recognizes a higher percentage of sentences. However, in ATC scenarios, ConER and CmdER are commonly used instead of *SER* due to high safety requirements.

The *F*1 *score* is a metric used to assess the performance of recognition models, used in conjunction with recall and precision, providing a comprehensive insight into performance. *Recall* measures the proportion of actual positive cases correctly identified by the model. It focuses on the model’s ability to capture true positives among all actual positives. It is typically calculated using the following formula:

(14) $$Recall=\frac{TP}{TP+FN}$$

where TP represents the correctly identified positive cases, and FN represents the positive cases missed by the model. Precision measures the proportion of correct positive predictions made by the model. It focuses on the model’s accuracy in labeling samples as positive. It is calculated using the following formula:

(15) $$Precision=\frac{TP}{TP+FP}$$

where FP represents the negative cases incorrectly labeled as positive. The *F*1 *score* is a metric that combines both *recall* and *precision* to provide a balanced assessment of a model’s performance. It is the harmonic mean of *recall* and *precision*, and it is calculated using the following formula:

(16) $$F1\text{-}score=\frac{2\times Recall\times Precision}{Recall+Precision}$$

The *F*1 *score* ranges between 0 and 1, with higher values indicating a better overall performance in terms of both coverage and accuracy.

*RTF* is used to measure the effectiveness of the ASR system, which is calculated as follows:

(17) $$RTF=\frac{T_{d}}{D_{s}}$$

where $T_{d}$ and $D_{s}$ are the calculation time and duration of the ATC speech, respectively.

In the ATC domain, the accurate identification of concepts takes precedence over the exact recognition of every word [24]. The ConER metric offers a keyword-focused evaluation approach that concentrates exclusively on the keywords within a given utterance. Concepts are formed by including the callsign information or the remaining command elements. For instance, in the utterance, “Good morning Air China one two tree climb level one two zero”, the concept “CCA123 CLIMB FL 120” is extracted, and the ConER quantifies this metric. The CmdER metric is a binary measure that checks the correctness of the entire sequence of concepts, similar to the commonly used SER.

To provide a clearer understanding, take the example of “DLH24F TURN LEFT HEADING 320” for better intuition. This recognition hypothesis has two concepts: “DLH24F” and “TURN LEFT HEADING 320”. In case the ATCo said, “Hello Lufthansa two four four turn left heading three two zero”, the ConER score for this statement would be 50%, indicating that half of the recognized concepts match the expected concepts. In contrast, the CmdER score would be 100%. In the context of ATC applications, ConER and CmdER metrics prove particularly useful for assessing ASR feedback on the relevant portions of an utterance for the planning system. Recognizing errors in non-ATC contexts is comparatively less critical.

**Figure 8:**
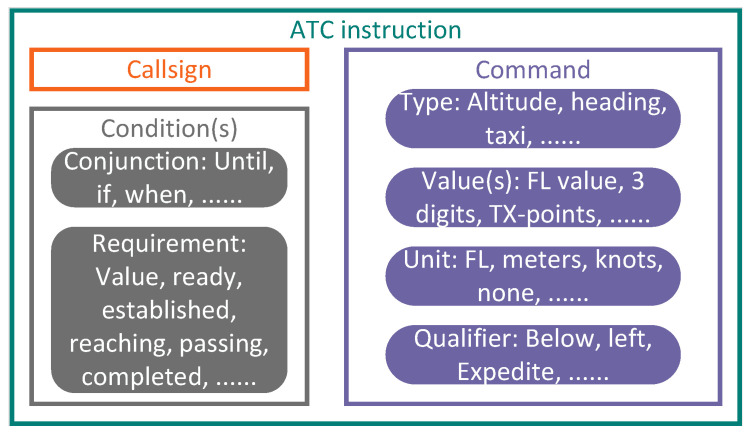
Elements of an ATC instruction.

While both ConER and CmdER metrics consider ATC-related factors, it is essential to conduct a comprehensive analysis and categorization of ATC concepts. The CWP HMI project has proposed an ontology for transcribing ATC instructions, defining abstract concepts related to ATC instructions and their interrelationships [45]. The critical components of ATC instructions consist of the callsign, command and condition, as shown in Figure 8. Each ATC instruction includes a command and one or more optional conditions. The command itself includes a type, one or more associated values and a unit of measurement. Additionally, an optional qualifier may also be included. It is important to note that a conditional clearance granted by an ATC officer only takes effect if specific requirements have been fulfilled. This ontology provides a detailed decomposition of ATC instructions, enlightening for applying ASR in the ATC domain. However, it has not been reflected in evaluating ASR systems so far.

The accurate recognition of callsigns holds paramount significance within ATC. It is crucial that all relevant details, including the airline name and flight number, are precisely identified to guarantee valid and efficient outcomes. The calculation of *CSA* is shown below:

(18) $$CSA=\frac{C_{Callsigns}}{T_{Utterances}}$$

The variables $C_{Callsigns}$ and $T_{Utterances}$ indicate the count of accurately identified callsigns and the total number of utterances in the test dataset, respectively.

Sichuan University’s research led to the development of specialized measures tailored to the ATC domain. The multilingual recognition performance in the ATC domain was considered, evaluating the ASR system by constructing indicators for Chinese characters and English words [54,61]. Additionally, to evaluate how well the ASR system performs during various control phases, the WERs for recognizing ground, tower and area speech were separately counted, facilitating the analysis of details [58]. Furthermore, the efficacy of the ASR approach was validated through its generalization and subsequent testing across various open corpora, thereby rendering it well-suited for real-time implementation in support of ATC applications [57,59].

Certain researchers have put forth evaluation metrics for ASR systems that are tailored to specific application objectives. The *RIA* measures the accuracy of repeating intentions and is calculated as follows:

(19) $$RIA=\frac{C_{intents}}{T_{Utterances}}$$

where $C_{intents}$ represents the number of utterances whose intents are correctly recognized [62]. Acceptable detection rates include the word detection rate, event detection rate and false positive rate, which are used to evaluate the application performance of commercial or open-source ASR software in real ATC communication [22,23]. The workload measurements show how much extra work ATCos can handle on top of their preparatory work [29,30].

While specific ATC-specific criteria exist, evaluating ASR systems within the ATC domain predominantly relies on generic metrics. Despite the proposal of an ontology for ATC instruction transcription, there is a notable absence of corresponding evaluation metrics, which is not facilitative to the targeted improvement of the ASR system. Therefore, it is necessary to improve the evaluation architecture to improve the performance of the ASR system in response to specific issues in the ATC domain. An evaluation framework is proposed in this review, as detailed in the next section.

### 3.5. Application Scenarios

Due to its promising potential for application in the ATC domain, ASR is currently being adopted across multiple ATC scenarios. These scenarios encompass ATC automation system integration, workload measurement and reduction for ATCos, callsign detection, read-back checking, pseudo-pilot, speaker role identification and command prediction, as shown in Table 5.

The AcListant and MALORCA projects aim to integrate ASR systems into the ATC automation systems to enhance overall ATC efficiency. In the AcListant project, the implementation of speech recognition served to streamline air traffic management, benefiting both ATCos and the AMAN system. The incorporation of ASR technology within the ATC environment improved the utilization of AMAN, providing ATCos with timely and consistent support while reducing the reliance on manual inputs. This, in turn, resulted in enhanced system efficiency and accuracy. The MALORCA project is initiated to automate the adaptation process of AcListant, replacing the resource-intensive manual adaptation. As part of this project, a series of machine learning mechanisms were developed to facilitate the seamless and automatic adaptation of AcListant to specific environmental conditions.

Irrespective of the original motivations behind ASR research, the ultimate objective is to enhance flight safety and operational efficiency. ATCo workload is identified as one of the main limiting factors to increasing overall system capacity and adequately matching it to demand. ASR technology in the ATC domain plays a pivotal role in automating the transcription of communication and effectively detecting control events executed by ATCos using voice commands. This, in turn, allows for the measurement of ATCos’ workload. An encompassing efficiency metric, which amalgamates throughput, flown distance, flight time and the availability of radar label information, has been defined [30]. The implementation of an ASR system brings significant relief to ATCos by reducing the time required for command input, minimizing discrepancies, and curbing the time spent rectifying radar label clearances. This not only streamlines their workload but also frees up cognitive resources, enabling them to handle a more substantial number of consecutive ATC commands. By harnessing the advantages of ASR technology, ATCos can enhance their operational efficiency and deliver a more efficient and effective air traffic management experience [29].

A callsign serves as a distinctive identifier for aircrafts, typically comprising the airline name’s abbreviation as its first part and the flight number as the last part. This callsign information constitutes critical data for ATC commands, demanding a high level of recognition accuracy. The exploration and implementation of advanced callsign detection technology can significantly reduce the workload of ATCos and enhance ATC safety. Typically, callsign detection implementations incorporate contextual information, such as radar or ADS-B data. This approach offers dual benefits: it enhances the accuracy of callsign recognition and verifies the correctness of the callsign voice by cross-referencing it with contextual information [66,72,74].

Following receipt of a command from an ATCo confirming with the pilot that the command has been accurately conveyed and comprehended is a crucial step, achieved through a read-back check. Read-back checking employs two primary methods: semantic matching [37] and ASR [58,63,70]. ASR-based read-back checking demands a high accuracy, a low rate of false alarms, and near real-time availability. Despite the seemingly straightforward nature of the read-back process, it has diversity and complexity in ASR tasks. Transcription and annotation effectively improve read-back checking performance in ASR tasks [70].

**Figure 9:**
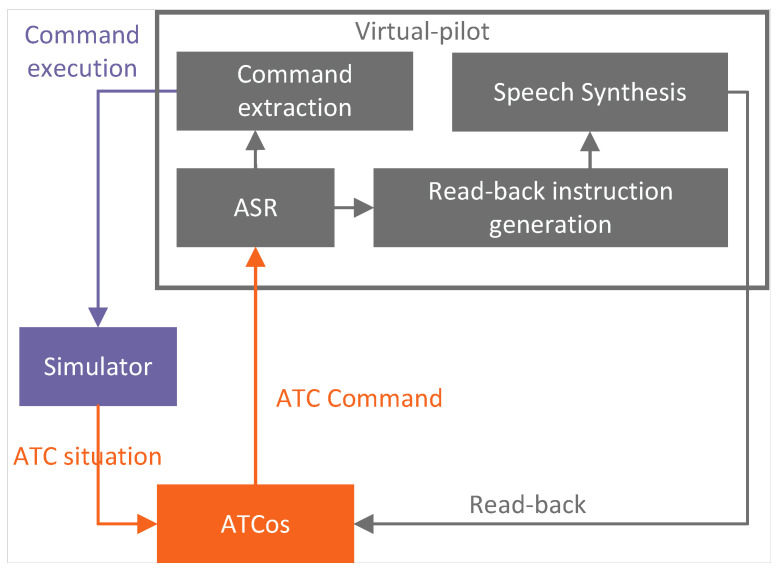
The autonomous training process for ATCos.

ATCos undergo rigorous training using simulation devices to acquire the qualifications necessary for their demanding roles in a realistic ATC environment. This training necessitates the involvement of a dedicated individual, often referred to as a pseudo-pilot, who simulates operational scenarios, resulting in additional training costs. The autonomous training process for ATCos is shown in Figure 9. An ATCo’s issued command is first converted into text using the ASR module. Subsequently, a text-based read-back instruction is generated. This text-based read-back instruction is then synthesized into speech and conveyed to the ATCo. Finally, the ATC command is executed within the simulator upon extraction. This comprehensive training approach ensures that ATCos are well-prepared to excel in their critical roles within the ATC domain.

Identifying the speaker’s role involves answering the fundamental question of “Who spoke when?” This process comprises several crucial components, including speech activity detection, segmentation or speaker change detection, embedding extraction, clustering, and labeling. On the other hand, command prediction entails generating hypotheses about the commands that the ATCo will likely issue soon. The utilization of an ASR system with command prediction capabilities leads to a significant reduction in the command recognition error rate [68].

An ASR software is developed by Sichuan University and used in real-world ATC scenarios [91]. It serves as a cornerstone for a wide range of critical applications within the ATC domain, offering substantial benefits to safety and operational efficiency. The core applications of this new system include normative instruction confirmation, aircrew read-back confirmation, conformance between ATCo intent and actual flight behaviors, potential conflict detection, runway incursion alert, similar callsign alert, speech rate measurement, and scene data storage. This ASR software presents a comprehensive suite of applications that have the potential to significantly bolster traffic safety, reduce workload burdens on ATCos, and proactively identify and address potential risks in ATC operations.

The gradual application of ASR in the ATC domain is of great help to the work of ATCos. By automating conventional communication tasks, ASR enables ATCos to focus on more critical air traffic management tasks. This not only improves efficiency but also helps reduce human errors caused by fatigue and information overload. However, the application of ASR may also have some negative effects on ATCos. When the ASR system cannot accurately recognize voice commands, ATCos need to spend more time and effort correcting these errors, increasing their workload and pressure. ASR systems perform differently in various noise environments and accents, which may introduce uncertainty and reduce ATCos’ trust in the system, thereby affecting their work efficiency and decision accuracy. On the other hand, overreliance on ASR technology may lead to the degradation of ATCos’ skills, especially in handling non-standard speech or unexpected situations.

In summary, although ASR offers significant advantages in the ATC domain, its application must be cautious to ensure it effectively reduces ATCos’ workload rather than adding new challenges. For instance, ASR must adapt to local accents and speech patterns to operate effectively. ASR should also possess error handling capabilities and provide mechanisms for ATCos to quickly correct errors. Additionally, appropriate training is essential to ensure ATCos can proficiently use ASR and fully harness its potential.

## 4. Evaluation Framework and Measurement for ASR Systems in the ATC Domain

In this section, we establish an evaluation methodology for ASR systems within the ATC domain. This methodology comprises a designated test corpus and a comprehensive evaluation framework. Evaluating the performance of an ASR system involves collecting recognition results generated by the ASR system within the test corpus and subjecting them to assessment through the evaluation framework. By meticulously analyzing the outcomes of this evaluation, ASR developers gain invaluable insights that empower them to implement precise modifications and enhancements, ultimately elevating the performance of the ASR system in the ATC domain.

### 4.1. Test Corpus for Evaluation

The choice of a test corpus for evaluating ASR systems is of paramount importance. It must be representative and diverse, encompassing various speech sources, speakers, pronunciations, and environmental conditions. This ensures that evaluations are based on real-world diversity and authenticity rather than being limited to specific scenarios. Moreover, the test corpus should be designed for repeatability, allowing other researchers or labs to replicate and validate the evaluation results. Additionally, it should cover the specific domain requirements, such as including relevant ATC instructions, flight numbers, and airline names in the case of ATC evaluations. The test corpus serves as the foundation for defining performance metrics and conducting comprehensive error analysis, providing valuable insights for system improvements. Ultimately, it empowers ASR developers to enhance system performance and meet the demands of specific domains.

To construct a comprehensive test corpus, it is imperative to encompass all elements of ATC speech that faithfully represent the structure and characteristics of ATC instructions and discourse. The categorization of the test corpus consists of the control phase, noise level, speech rate, control area, and gender, with each speech belonging to only one category in each classification. Taking China’s ATC as an illustration, according to the approximate proportion of ATC commands, the proportion of each category in the test corpus aligns with the distribution of ATC commands, as depicted in Table 6. Control areas are divided because of the varying accents and waypoints found in different regions. In this section, the test corpus is divided equally according to the structure of Chinese civil aviation and includes English with Chinese accents as additional material.

To comprehensively evaluate the ASR systems in the ATC domain, each speech in the test corpus is annotated with a three-level label. At the first level, a full-text annotation is applied to each speech, providing a general ASR annotation that facilitates the calculation of word and sentence error rates compared to the recognized text. The second-level label involves text classification, categorizing different segments of an ATC command, such as callsign, command type, command content, meteorological information, identification and transfer, fixed-name information, other key information and miscellaneous content. This classification serves the purpose of evaluating ASR performance on distinct components of ATC commands, providing insights into recognition accuracy for various ATC command elements. The third level of annotation includes auxiliary labels indicating the control phase, noise level, speech rate, control area and speaker gender. Adding these labels allows for a deeper evaluation of the tested ASR system’s performance in specific categories, guiding developers in making targeted improvements. This three-level labeling system ensures a comprehensive evaluation of ASR systems in the ATC domain, covering various aspects of recognition accuracy and providing valuable insights for system enhancement.

### 4.2. Evaluation Framework

**Figure 10:**
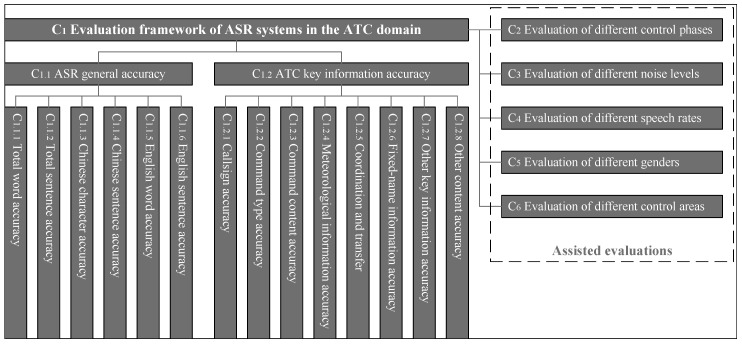
Evaluation framework of ASR systems in the ATC domain.

The evaluation framework comprehensively evaluates an ASR in the ATC domain. It serves as a multifaceted tool for analyzing the performance of these systems across various dimensions, ultimately guiding their enhancement. This evaluation framework includes three key components: ASR general accuracy, which gauges the system’s overall recognition performance; ATC key information accuracy, ensuring the correct understanding of vital details; and assisted evaluations that provide insights from diverse perspectives. To gain a visual understanding of this framework, refer to Figure 10.

ASR general accuracy measures the recognition of an entire speech, excluding the evaluation of ATC-specific content recognition. This metric evaluates the precision of recognizing words and sentences in multilingual contexts. English is the prescribed language for ground–air communication in international flights, while native languages are permissible for domestic flights. Due to variations in grammar and pronunciation among different languages, it is advisable to evaluate recognition results separately for a more comprehensive understanding. For instance, in the context of ATC operations in China, ASR accuracy comprises assessments of total word accuracy, total sentence accuracy, English word accuracy, English sentence accuracy, Chinese character accuracy and Chinese sentence accuracy.

A typical ATC utterance comprises crucial information, such as callsigns, and non-critical elements, like “hello” or “goodbye”. Ensuring the complete and accurate recognition of all key information within an ATC utterance is imperative, with even minor deviations from expected recognition considered as failures. Presently, ASR research in the ATC domain primarily focuses on recognizing callsigns, emphasizing the importance of achieving a high recognition accuracy for this critical element. Notably, in the CWP HMI project, an ontology was proposed to facilitate the translation of ATC speech commands [45]. However, the key information of ATC has not yet been integrated with ASR.

Different components within ATC commands exhibit distinct characteristics in terms of semantics and pronunciation. Evaluating the recognition performance of ASR for each component individually is valuable for developers seeking targeted improvements. In this context, we categorize these evaluation indicators into specific components, including callsign, command type, command content, meteorological information, coordination and transfer, fixed-name information, other key information and other content. This categorization enables a more precise assessment of ASR systems’ recognition capabilities for various ATC key information elements, facilitating performance enhancement efforts. The command type refers to ATC commands such as taxi, approach, departure, altitude adjustment, speed adjustment, heading adjustment, etc. The command content refers to the adjusted values and units under the command type. In ground–air communication, meteorological terms usually differ from other communication terms and are listed separately for recognition accuracy evaluation. Fixed-name information includes the airport, aircraft type, route and waypoint, which can be queried in the information database for direct matching.

ATC speeches can also be classified according to the control phase, noise level, speech rate, gender and control area, each exhibiting distinct characteristics. When considering the control phase, ground control involves clearance and taxiing commands, tower control handles takeoff and landing commands, while the approach and area phases primarily deal with aircraft maneuver commands. Classification according to noise level or speech rate reveals variations in ASR system performance, with some systems excelling in low-noise and slow-speech scenarios but struggling in high-noise or rapid-speech situations. Furthermore, differences in pronunciation characteristics between male and female ATCos may impact ASR system performance. Not all ATCos use standard Mandarin in different control areas, and various regional accents may be present. It is essential to conduct evaluations based on control areas. These assessments offer insights into the ASR system’s strengths and weaknesses, allowing for a more comprehensive analysis beyond just ASR accuracy evaluation.

ASR general accuracy serves as a metric to gauge ASR performance, while ATC key information accuracy dissects the speech into ATC elements, evaluating their recognition performance individually. Assisted classification indicators assess recognition performance based on non-speech attributes of ATC speech, yielding more nuanced evaluation results. Employing these three categories of metrics for ASR system evaluation offers a more comprehensive approach than conventional methods.

## 5. Discussion

### 5.1. Challenges of ASR in the ATC Domain

While ASR in the ATC domain has achieved noteworthy milestones, its widespread adoption in real ATC scenarios remains challenging. This section expands upon the challenges identified in previous studies [7,10], including unsatisfactory recognition accuracy, the extra workload for ATCos, non-standard speech, incomplete corpus, poor generalization, unprofessional evaluation methods, impact on current ATC operations and some ASR technical issues.

- Safety stands as a paramount criterion in ATC operations. Despite achieving an impressive ASR recognition accuracy of approximately 96%, there remains a residual error rate of around 4%. In real ATC scenarios, such error rates can foster distrust in ASR among ATCos. On the one hand, they must contend with correcting recognition errors, and on the other hand, they also need to verify accurate recognition. This not only fails to reduce ATCos’ workload but also adds to their burdens.
- One of the purposes of implementing ASR is to reduce the workload of ATCos, particularly when integrated with ATC automation systems. However, recognition errors can result in either missed alarms or false alarms. Missed alarms pose safety hazards to aircraft operations, while false alarms place an additional burden on ATCos.
- In real ATC scenarios, not all sentences spoken by ATCos adhere to standardized protocols. This is particularly evident during emergencies, where ASR systems may struggle to recognize and interpret ATCo communications accurately.
- The quality of the ATC corpus significantly affects ASR performance, making it challenging to collect sufficient training samples to develop a capable ASR system. Annotating the training samples for ASR in the ATC domain requires significant expertise and knowledge of ATC principles. Such expertise may only be readily available to some staff, and specialized training may be necessary to ensure competence in this area.
- Generalization is significant to ASR research in the ATC domain. It is essential to recognize that the vocabularies used in different control centers or locations may exhibit unique and distinct characteristics. Consequently, enhancing the generalization capabilities of ASR systems across diverse control centers or areas becomes a crucial technique for broadening the applicability of ASR technology.
- The use of ASR will have an impact on existing ATC operations. The ASR system needs to consider effective interaction with ATCos during design, including voice command confirmation, error correction and system feedback, in order to improve user experience and operational efficiency. Therefore, the introduction of ASR systems may require adjustments and redesign of existing operational processes to adapt to the use of speech recognition technology. ATCos needs to receive training and an adaptation period on new technologies to proficiently use the ASR system and understand its limitations and correct usage methods.

In addition, the ATC domain presents several common challenges, including noise interference, unstable speed rates, multilingual speech, accented speech, code-switching, etc., which will not be reiterated in this article.

### 5.2. Approaches to Improve ASR Performance

In response to the challenges faced, the following approaches can be applied to improve the performance of ASR in the ATC domain.

- To ensure consistency between label text and recognition text, and to accurately represent the intent of ATCos in the recognized text, it is essential to establish design specifications for data annotation and transcription. This standardization plays a crucial role in minimizing recognition errors attributable to non-technical factors. For example, should “Air China 4137” be labeled as “CCA 4137” or “AIR CHINA FOUR ONE THREE SEVEN”.
- Training samples should be thoughtfully chosen to reasonably cover various ATC scenarios, focusing on different control phases, control areas, speech rates, and noise levels. An ASR system trained using a corpus with well-balanced coverage and standardized annotation can significantly enhance its recognition performance in real-world ATC scenarios.
- To tackle the challenge of limited generalization, it is crucial to establish an information database that encompasses control units, airline information, waypoints from various control areas, and route names. During the ASR process, the recognized text can be cross-referenced with the information stored in the database. This approach enhances the generalization capabilities of ASR systems, allowing systems trained for one control area to be effectively utilized in other regions as well.
- Choosing appropriate metrics and developing an evaluation framework aligned with specific application requirements is a crucial step. This process aids in pinpointing shortcomings in ASR performance and facilitates necessary adjustments and improvements. For example, although the lower the WER or SER, the better, considering the limitations of current technological means, they should be set at an achievable and reasonable level. The remaining measures should be selected and set according to actual application requirements.
- In specific ATC speech scenarios, the content can become ambiguous and challenging to recognize accurately without proper context. Integrating contextual information, including system-level integration with surveillance systems and ATC automation systems, as well as ASR-level integration with pre-speech data, can enhance the semantic understanding and intention recognition of ATC speech.
- Implementing a graded alarm mechanism is crucial for minimizing false alarms and missed alarms. In actual ATC scenarios, using different levels of alarms for different types and degrees of risks can enable ATCos to effectively allocate attention and resources, thereby reducing security risks and preventing additional workloads.
- Although not used in the ATC domain, audiovisual speech recognition (AVSR) has shown potential. Currently, ATCos operate in a video surveillance environment, so a combination of audio and video can be used to improve recognition accuracy. In noisy and multiplayer environments, visual signals, such as lip-reading signals, can provide additional sources of information and enhance speech recognition capabilities. When using AVSR in the ATC domain, attention should be paid to issues such as data security and privacy, system complexity and increased costs, real-time performance, and audio–video synchronization [92,93,94].
- With the development of big data, artificial intelligence, E2E and other advanced technologies, ASR technology is evolving and improving. It is imperative to stay current with these advancements and incorporate advanced ASR technology into the ATC domain to enhance overall performance.
- Expanding the functions related to human factors is crucial for improving ASR performance. By analyzing the speech characteristics of ATCos, ASR systems can detect fatigue, stress and emotions, issue timely warnings and help manage work tasks. Conducting ASR trials and training with ATCos provides feedback for improvement and enhances adaptability. This approach not only boosts ASR performance but also ensures better integration into real-world ATC environments.

## 6. Conclusions

ASR has been studied in the ATC domain for many years and has achieved fruitful achievements. This review presented a thorough overview of the current research on ASR in the ATC domain. We delved into various contributions, encompassing aspects such as corpora, models and their extensions, evaluation measures and application scenarios. Moreover, we analyzed the evaluation metrics for ASR in the ATC domain and constructed an evaluation framework. Additionally, this review further elucidated the challenges faced while implementing ASR technology in realistic ATC scenarios and provided valuable insights for future research and development initiatives.

The applicability of ASR systems in ATC scenarios is still an area of ongoing exploration. Nevertheless, it is evident that continued advancements in the ASR field hold the potential to overcome these challenges in the near future. This review intends to provide inspiration to researchers and promote further developments in ASR research within the ATC domain. We anticipate that our findings will contribute to fruitful outcomes in future research endeavors.

## Figures and Tables

**Table 1:** Automatic speech recognition in air traffic control.

Papers	Technique Details	Research Groups or Projects
[18]	Construction of the ATCOSIM corpus	Eurocontrol
[19]	HMM and cross-task adaptation	Polytechnic University of Madrid
[20]	Construction of a Spanish and an English corpora separately	Polytechnic University of Madrid
[21]	HMM and PPRLM, construction of corpora for tower control	Polytechnic University of Madrid
[22,23]	Human interface, callsign recognition, reduction of ATCos’s workload	CRIDA
[24]	Combination of AMAN and ASR	AcListant
[25]	Construction and annotation of corpora	AcListant
[26]	Construction of a simulated corpus with German and Czech accents	AcListant
[27]	ABSR, improvement of the corpus to distinguish between male and female	AcListant
[28,29,30]	Demonstration of reducing the workload of ATCos	AcListant
[31]	Instruction error correction for ATCos	FAA
[32]	Semantic understanding to reduce WER	Østfold University College
[33]	Proposal of the pseudo-pilot concept	Optimal Synthesis
[34]	Publication of the AIRBUS-ATC corpus	AirBus
[35]	A challenge competition	AirBus
[36]	Proposal of an advanced ASR architecture for ATC	AirBus
[37]	Repetition detection matching	Civil Aviation University of China
[38]	DNN-HMM	Civil Aviation University of China
[39]	Construction of the MALORCA corpus	MALORCA
[40]	Proposal of the command prediction model	MALORCA
[41]	Semi-supervised training to expand unlabeled data	MALORCA
[42]	The generalization of ASR	MALORCA
[43]	Use of domain knowledge	MALORCA
[44]	Use of iterative methods to utilize unannotated data	MALORCA
[45]	Proposal of an ontology for data transcription	CWP HMI
[46]	Use of a commercial framework for easy implementation	CWP HMI
[47]	Proposal of the command extraction model	CWP HMI
[48]	Proposal of a method to reduce speech rate	Nanyang Technological University
[49]	Combination with commercial software	Robust Analytics
[50,51]	Proposal of a comprehensive method for constructing a corpus	University of West Bohemia
[52]	Use of an end-to-end ASR model	NUAA
[53]	Use of a cascading model to solve the multilingual problem	Sichuan University
[54]	Proposal of a method for feature extraction	Sichuan University
[55]	Use of one model to recognize both Chinese and English languages	Sichuan University
[56]	Construction of the ATCSpeech corpus	Sichuan University
[57]	Optimization of the language model	Sichuan University
[58]	Use of unsupervised pretraining and transfer learning	Sichuan University
[59]	Combination of self supervision, unsupervised learning and supervised learning	Sichuan University
[60]	Proposal of a comprehensive system	Sichuan University
[61]	Proposal of a language model containing callsigns	Sichuan University
[62]	Use of a residual network	Sichuan University
[63]	Proposal of a method for multitasking learning	Sichuan University
[64]	Use of multiple corpora	MIT
[65]	Use of a hybrid model and LF-MMI	ATCO2, HAAWAII
[66]	Callsign recognition	ATCO2
[67]	Tower command recognition	HAAWAII
[68]	Remote tower	HAAWAII
[69]	Added a pilot role in the speech	HAAWAII
[70]	Evaluation indicators with repetition recognition	HAAWAII
[71]	Multi-task AM	HAAWAII
[72]	Decoder	ATCO2, HAAWAII
[73]	Use of semi-supervised learning	ATCO2, HAAWAII
[74]	Data annotation	ATCO2
[75]	Proposal of a comprehensive model with contextual information	ATCO2
[76]	Data augmentation and BERT	ATCO2
[77]	Callsign recognition with contextual information	ATCO2
[78]	Discussion of the amount of data required for ASR in ATC	ATCO2, HAAWAII
[79]	Proposal of a hybrid model combining CNN, TDNN and LM	ATCO2, HAAWAII
[80]	Data augmentation and BERT	ATCO2, HAAWAII
[81]	Construction of the ATCO2 corpus	ATCO2, HAAWAII

**Table 2:** Corpora of ASR in the air traffic control domain.

Corpora	Language and Accent	Real/Simulated	ATC Phase	Data Size	Used in Papers
LDC94S14A	US, English	Real	Tower, approach	72.5 h	[64,65,66,78,80]
N4 NATO	Canadian, German, Dutch and British-accented English	Simulated	Military	9.5 h	[66,73]
HIWIRE	French, Greek, Italian and Spanish-accented English	Simulated	Military	28.3 h	[65,66,73]
Madrid airport	Spanish, English	Real	Ground, tower	11.8 h	[19,20,21]
Madrid ACC	Spanish, English	Real	Approach, en-route	100 h	[22,23]
ATCOSIM	German, Swiss, and French accented English	Simulated	En-route	10.7 h	[18,33,50,64,65,66,73,78]
AcListant	German and Czech-accented English	Simulated	Approach	8 h	[24,25,26,27,28,29,30]
DataComm	US, English	Simulated	Tower, approach, en-route	144 × 50 min	[31]
ATCSC	English	Simulated	Clearance	4800 utterances	[32]
AIRBUS-ATC	French-accented English	Real	Tower, Approach, ATIS	59 h	[34,35,36,64,65,66,74,77]
MALORCA	German and Czech-accented English	Simulated	Approach	10.9 h	[39,40,41,42,43,44,46,47,65,66,72,74,76,77,79]
UWB-ATCC	Czech-accented English	Real	Tower, approach, en-route	179 h	[50,51,64,65,66,78]
SOL-Twr	Lithuanian-accented English	Real	Tower	1993 utterances	[67]
SOL-Cnt	German-accented English	Real	Approach	800 utterances	[76,80]
HAAWAII	Icelandic and British-accented English	Real	Tower, approach, en-route	34 h	[69,70,76,78,79,80]
ATCO2	English	Real	Ground, tower, approach, en-route	4+5281 h	[71,75,76,78,80,81]
ATCSpeech	Mandarin Chinese, English	Real	Ground, tower, approach, en-route	58 h	[53,54,55,56,57,58,59,60,61,62,63]
LiveATC		Real	Ground, tower, approach, en-route		[50,71,72,74,75,77,78,79,80]

**Table 3:** ASR model in the ATC domain.

Papers	Feature Extraction	Acoustic Model	Language Model	Decoder
[19]	CMVN	HMM	\	\
[20,21]	CMVN	HMM	Stochastic bigram LM	\
[22,23]	\	HMM	Grammar LM	\
[24]	\	GMM-HMM	\	WFST
[25]	\	GMM-HMM	Grammar LM	WFST
[26]	\	GMM-HMM	Grammar LM	Context-dependent WFST
[27]	\	GMM-HMM	N-gram LM	WLD
[32]	\	Generic AM	N-gram LM	N-best
[33]	Non-specific	Generic AM	Not given	Non-specific
[34]	Non-specific	TDNN	4-gram LM	\
[35]	Multiple	Multiple	Multiple	Multiple
[36]	MFCC	BiLSTM	RNN LM	N-best
[38]	MFCC+CMVN	CDNN-HMM	Non-specific	\
[39]	\	DNN-HMM	3-gram LM,	Context-aware rescoring N-best
[40,42,44]	\	DNN-HMM	N-gram LM	\
[41]	\	DNN-HMM	3-gram LM	\
[43]	\	DNN-HMM	3-gram LM	Domain knowledge-based N-best
[47]	\	DNN-HMM	N-gram LM	Command extractor
[48]	STRAIGHT	\	\	\
[50]	\	DNN-HMM	3-gram LM, oracle	\
[51]	\	DNN-HMM	N-gram LM	\
[52]	\	E2E model (CNN+BiLSTM+CTC)	LSTM LM	CTC-decoder
[53]	MFCC	Cascade model (CNN+BiLSTM+CTC)	PLM+WLM, PM	CTC-decoder
[54]	CNN	E2E model (CNN+BiLSTM)	\	CTC-decoder
[55]	\	Hybrid model (CNN+BiLSTM)	RNN LM	CTC-decoder
[56]	\	DNN+CTC	N-gram LM	CTC-decoder
[57]	MFCC	Cascade model (MCNN/AP+BLSTM+CTC)	RNN LM(PLM+WLM), MTPM	Generic decoder
[58,59,60]	MFCC	E2E model (MCNN/AP+BLSTM+CTC)	\	CTC-decoder
[61]	\	E2E model (CNN+RNN)	Context-aware LM	Context-aware decoder
[62]	HFE	E2E model (CNN+RNN)	\	CTC-decoder
[64]	MFCC	RNN+CTC	N-gram	Generic decoder
[65]	\	TDNNF	SRI trained N-gram	\
[66]	\	TDNN	N-gram	WFST
[67]	\	CNN+TDNNF and DNN-HMM	N-gram	\
[70,71]	\	CNN+TDNNF	3-gram	\
[72]	MFCC	CNN+TDNNF	3-gram	\
[73]	MFCC	CNN+TDNNF	3-gram	WFST
[74]	MFCC	CNN+TDNNF	Context-aware 3-gram	WFST
[75]	MFCC+CMN	CNN+TDNNF	3-gram	WFST
[78]	\	E2E model	\	\
[79]	MFCC	CNN+TDNNF	4-gram	Context-aware WFST
[80,81]	MFCC	CNN+TDNNF	3-gram	WFST

**Table 4:** Evaluation measures for ASR in the ATC domain.

Measure Classification	Evaluation Measure	Used in Papers
General ASR measure	Word/character accuracy/error rate	[19,20,21,24,25,26,27,29,32,35,36,38,39,41,42,43,44,51,52,53,54,55,56,57,58,61,62,63,64,65,66,67,71,72,73,74,75,77,78,79]
	Sentence accuracy/error rate	[20,21,24,52]
	F1 score	[35,36,53,55,63,66,75,76]
	Real-time factor	[26,38,39,53]
ATC key information measure	Concept error rate	[24,26,27,39,41,42,43,64]
	Command error rate	[25,26,27,29,39,44,46,67]
	Callsign accuracy	[61,63,67,70,72,73,74,75,79]
Application measure	Repetition intent accuracy	[55,63]
	Acceptable detection rate	[22,23,31]
	Workload measurements	[29,30]

**Table 5:** Application scenarios of ASR systems in the ATC domain.

Application	Used in Papers
ATC automation system integration	[24,25,26,27,28,29,30,39,40,41,42,43,44]
Measurement and reduction of workload for ATCos	[22,23,29,30,46]
Callsign detection	[22,23,35,36,61,66,72,73,74,75,77]
Read-back checking	[37,55,63,70]
Pseudo-pilot	[33,51,60,63]
Speaker role identification	[71,75,76,80]
Command prediction	[42,43,44,68]

**Table 6:** Composition of the test corpus.

Classification	Items	Proportion
Control phase	Ground	8%
	Tower	30%
	Approach	30%
	Area	30%
	Emergency	2%
Noise	Clean	20%
	Low noise	50%
	High noise	30%
Speech rate	Slow	20%
	Normal	40%
	Fast	40%
Control area	Northeast China	13%
	North China	13%
	Northwest China	13%
	East China	13%
	Southwest China	13%
	Central and South China	13%
	Xinjiang, China	13%
	Chinese-accent English	9%
Gender	Male	70%
	Female	30%

## Data Availability

We conducted experiments using Chinese speech data to verify the rationality of the proposed test corpus composition and evaluation method. Detailed experimental reports are available from the corresponding author upon reasonable request.

## Abbreviations

The following abbreviations are used in this manuscript:

ABSR	Assistant-based speech recognition
AED	Attention-based encoder–decoder
AM	Acoustic model
AMAN	Arrival manager
AP	Average pooling
ASR	Automatic speech recognition
ATC	Air traffic control
ATCos	Air traffic controllers
BERT	Bidirectional encoder representations from transformers
CER	Character error rate
CMVN	Cepstral mean and variance normalization
CNNs	Convolutional neural networks
CmdER	Command error rate
ConER	Concept error rate
CSA	Callsign accuracy
CTC	Connectionist temporal classification
DNN	Deep neural networks
E2E	End-to-end
FAA	Federal Aviation Administration
FBank	Filter bank
FC	Fully connected
GMM	Gaussian mixture model
HMM	Hidden Markov model
ICAO	International Civil Aviation Organization
LF-MMI	Lattice-free maximum mutual information
LM	Language model
LSTM	Long short-term memory
MCNN	Multiscale CNN
MFCC	Mel frequency cepstrum coefficient
MTPM	Machine translation PM
PLM	Phoneme-based LM
PM	Pronunciation model
PPR	Parallel phone recognition
RIA	Repetition intention accuracy
RNNs	Recurrent neural networks
RNN-T	Recurrent neural network transducer
RTF	Real-time factor
SER	Sentence error rate
STFT	Short-time Fourier transform
TDNN	Time delay neural network
VHF	Very high frequency
WER	Word error rate
WFST	Weighted finite-state transducer
WLM	Word-based language model
